# Circular RNA DGKB Promotes the Progression of Neuroblastoma by Targeting miR-873/GLI1 Axis

**DOI:** 10.3389/fonc.2020.01104

**Published:** 2020-07-23

**Authors:** Jiale Yang, Leitao Yu, Jinlong Yan, Yu Xiao, Weiming Li, Juhua Xiao, Jun Lei, Deng Xiang, Shouhua Zhang, Xin Yu

**Affiliations:** ^1^Department of General Surgery, Second Affiliated Hospital of Nanchang University, Nanchang, China; ^2^Department of General Surgery, Jiangxi Provincial Children's Hospital, Nanchang, China; ^3^Department of Ultrasound, Jiangxi Provincial Maternal and Child Health Hospital, Nanchang, China

**Keywords:** neuroblastoma, circDGKB, miR-873, GLI1, diagnostic marker

## Abstract

Accumulated evidences suggested that circular RNAs (circRNA) played critical roles in tumorigenesis and progression. To our knowledge, no study reported the function of circular RNA DGKB (circDGKB, circRNA ID: hsa_circ_0133622) on progression of neuroblastoma (NB). Here, we showed that circDGKB was upregulated in NB tissues compared to the normal dorsal root ganglia. Moreover, the expression level of circDGKB was negatively correlated with the survival rate of NB patients. Mechanically, overexpression of circDGKB promoted the proliferation, migration, invasion, and tumorigenesis of NB cells and reduced cell apoptosis, and vice versa. In addition, qRT-PCR and/or Western blot results showed that circDGKB overexpression inhibited the expression level of miR-873 and enhanced GLI1 expression. Moreover, miR-873 functioned an opposite role to circDGKB and significantly weakened circDGKB role in promoting NB progression. Furthermore, GLI1 upregulation also rescued the miR-873 role in inhibiting NB progression. In conclusion, our work proved that circDGKB promoted NB progression via targeting miR-873/GLI1 axis *in vitro* and *in vivo*. Our study provided a new target for NB treatment and indicated that circDGKB could act as a novel diagnostic marker for NB.

## Introduction

Neuroblastoma (NB) is a malignant tumor mainly occurring in children (1), which commonly arises in adrenal glands, and also occurs in the abdomen, neck, spine, or chest (2). The most malignant tumors have amplification of the MYCN oncogene encoding a transcription factor, which is associated with poor survival of NB, even in localized diseases (3). NB is divided into two classifications in clinic, low-risk disease and high-risk disease. Patients with low-risk disease respond positively to therapy and even can recover without any treatment. Unfortunately, 40% of NB patients suffer from high-risk disease which is easy to relapse and lethal (1, 4). Various kinds of therapeutic methods have been adopted to treat high-risk NB, including surgical treatment, chemotherapy, radiotherapy, immunotherapy, cis-retinoic acid, and proton therapy (5), but the prognosis of NB is still poor due to the complex mechanisms underlying NB occurrence and development. Therefore, it is urgent to further reveal the molecular mechanisms of NB in order to find novel and potent targets for the treatment of NB.

Circular RNAs (circRNAs) are a class of non-coding RNA molecules generated from exons or introns by forming covalently-closed loops (6). Previous studies have suggested that circRNAs can serve as the sponges of microRNAs (miRNAs) to inhibit the function of miRNAs. For example, circATP82A2 repressed the expression of miR-443 via binding to miR-443 directly (7). circRNAs are abundantly expressed in eukaryotic organisms and closely implicated in a variety of disease, such as diabetes, cardiovascular diseases (CVDs), and cancers (8, 9). Numerous studies demonstrated that circRNA/miRNA axis played critical roles in the progression of cancers. For instance, circ0103552 significantly promoted cell proliferation and invasion via sponging miR-1236 in breast cancer (10). Mao et al. (11) demonstrated that circ0068871 could accelerate the progression of bladder cancer by targeting miR-181a-5p and then regulate the expression of FGFR3 and activate STAT3. Huang et al. (12) identified that circ100338 was highly expressed in hepatocellular carcinoma (HCC) and functioned as an inhibitor of miR-141-3p to promote the proliferation of HCC cells. However, the functions of lots of circRNAs are waiting for us to explore.

MiRNAs are short non-coding transcripts with 22 nucleotides in length (13). Numbers of studies have demonstrated that miRNAs played significant roles in RNA translation and degradation via binding to the 3′UTR of mRNA (14). MiRNAs were proved to participate in progress of many kinds of cancers by acting as the targets of circRNAs and then regulating the expression of oncogenes or tumor suppressive genes (15). In the present study, we found that circDGKB (circRNA ID: hsa_circ_0133622) was highly expressed in NB. In addition, circDGKB could act as an oncogene in NB by targeting miR-873/GLI1 (Glioma-associated oncogene 1) axis. Our study provided a novel potential target for the diagnosis and treatment of NB in clinical.

## Materials and Methods

### Tissue Collection

Thirty NB tissues were obtained from 30 NB patients at Second Affiliated Hospital of Nanchang University during 2015 to 2017. Meanwhile, 10 human normal dorsal root ganglia were collected from interrupted pregnancies, which were used as a normal control group. Patients participated in this study never received any other therapeutic method but surgery and the informed consents were signed by their parents. This study was approved by the Research Ethics Committee of Second Affiliated Hospital of Nanchang University.

### circRNA Microarray

After being obtained from surgical specimens, 3 NB tissues and 3 normal dorsal root ganglia were immediately frozen using liquid nitrogen. Sample preparation and microarray hybridization were carried out in accordance with the protocols of Arraystar (Rockville, MD, USA). circRNAs were enriched through removing linear RNAs with Rnase R (Epicentre, Madison, WI, USA), followed by amplification and labeling with Arraystar Super RNA Labeling Kit (Arraystar). Then, the Arraystar Human circRNA Array (8 × 15 K) was used for hybridization and scanned by the Agilent Scanner G2505C (Jamul, CA, USA). circRNAs demonstrating fold-changes of ≥2 and *P*-values of < 0.05 were considered as significantly differentially expressed.

### Cell Culture

Two NB cell lines SK-N-SH and SH-SY5Y were bought from American Type Culture Collection (ATCC, VA, USA) and cultured in Dulbecco's Modified Eagle Medium (DMEM, Gibco, CA, USA), with 10% fetal serum (FBS, Gibco, CA, USA) and 1% penicillin/streptomycin (Invitrogen, CA, USA).

### Cell Infection

The lentiviral vectors used to upregulate circDGKB (Lentiv-circDGKB), miR-873 (mimic-miR-873), and GLI1 (Lentiv-GLI1) in SK-N-SH and SH-SY5Y, and the lentiviral vectors used to downregulate circDGKB (shRNA-circDGKB), miR-873 (inhibitor-miR-873), and GLI1 (shRNA-GLI1), in addition to the negative control vector (Lentiv-NC, inhibitor-NC, mimic-NC), were all designed and synthesized by GenePharma (Shanghai, China). These vectors were introduced into SK-N-SH and SH-SY5Y cells by cell infection with the help of polybrene (Hanbio, Inc, Shanghai, China), followed by incubation with G418 or puromycin for 14 days to establish the stable transfection cell lines used in *in vivo* experiments.

### Cell Growth Assay

Cell Counting Kit-8 (CCK-8) (Beyotime, Beijing, China) was adopted to assess the proliferation of NB cells. The cells were seeded into 96-well plates at the density of 2,000 cell/well and incubated at 37°C. Following 24 h, the cells were infected with lentiviral vectors instantaneously. Forty-eight hours later, 10 μl CCK-8 solution was added into each well and incubation at 37°C for another 2 h. The absorbance was detected by a plate reader (model 680; Bio-Rad, Hertfordshire, UK) at 450 nm.

### Cell Migration Assay

NB cells were seeded into six-well plates at the density of 3,000 cells/group. The wounds were scraped by pipettes following cell infection. The width of the wound was captured at 0 and 24 h following scratch by DM2500 bright field microscope (LEICA, Wetzlar, Germany) and the imageJ software was used to quantify the migration distance.

### Transwell Invasion Assay

The upper chamber of the transwell chambers (8 μm, BD Bioscience, USA) were first coated with matrigel. Then, the infected SK-N-SH and SH-SY5Y cells were suspended with FBS-free medium and seeded into the upper chamber at the density of 3,000 cells/group. At the same time, in the bottom chamber were added 600 μl complete medium containing 10% FBS (Gibco). Following 48 h of incubation at 37°C, the inversed cells were fixed with 4% paraformaldehyde and stained with crystal violet. The number of inversed cells from 6 randomly selected fields were counted.

### Cell Apoptosis Assay

Cell apoptosis detection was performed by using flow cytometry with the help of Annexin V-FITC/PI Apoptosis Detection Kit (Vazyme, Biotech, Jiangsu, China) according to the instructions of the manufacturer.

### Luciferase Reporter Assay

The wild type (circDGKB-WT) and mutated type (circDGKB-MUT) vectors between the binding sites in circDGKB were synthesized by GenePharma. Then, SK-N-SH cells were co-transfected with mimic-NC/mimic-miR-873 and circDGKB-WT/circDGKB-MUT using Lipofectamine (Invitrogen) or polybrene. Following 48 h, the relative luciferase activity was detected by Dual Luciferase Assay System (Promega, Madison, WI, USA) according to the manufacturer's instructions.

### Western Blot

The cells were lysed with RIPA lysis buffer (Thermo Fisher Scientific, NYC, USA) supplemented with protease inhibitor cocktail (APExBIO, AL, and USA) on ice. Then, the supernatant was transferred into new microphage tubes (Eppendorf, Hamburg, Germany), followed by centrifugation at 12,000 rpm for 30 min at 4°C. The concentrations of protein samples were measured with BCA Protein Quantification Kit (YEASEN, Shanghai, China). Next, 30 μg protein of each sample was used for electrophoresis. After transferring the protein to PDVF membrane, the membranes were blocked with 5% milk in TBST for 1 h. Primary antibodies included anti-ZBE1 antibody (No. ab203829, Abcam, Cambridge, MA, USA), anti-GLI1 (No. ab49314, Abcam), and anti-GAPDH antibody (No. ab011-040, Multi sciences, Zhejiang, China). The secondary antibodies was used as follows: anti-Mouse IgG-HRP (No. RA230188, Thermo Fisher Scientific).

### Reverse Real-Time Quantitative Polymerase Chain Reaction (qRT-PCR)

Total RNA extraction from cells and tissues was carried out with Trizol reagent (Invitrogen). Then, the RNAs were reversed into cDNAs by using the high capacity cDNA Reverse Transcription Kit (Applied Biosystems, Foster City, CA). TaqMan Universal Master MixII (Takara, Dalian, China) was adopted to perform real-time quantitative PCR and the internal reference was GAPDH/U6.

Primers are shown as follows:

circDGKB-forward: 5′-AGACTCTGCCCACTTCAGGA-3′,
circDGKB-reverse: 5′-AGGCACTGGGTCTCCTTTCT-3′;
miR-873-forward: 5′-TGTGCATTTGCAGGAACTTGT-3′,
miR-873-reverse: 5′-GGGAACTCATCAGTCTCCTGTTC-3′;
GAPDH-forward: 5′-CCACCCCCAATGTCTCTGTT-3′,
GAPDH-reverse: 5′-ATGGATGAACGGCAATCCCC-3′.

### *In vivo* Tumorigenicity Assay

Animal study was performed in accordance with the Research Ethics Committee of Second Affiliated Hospital of Nanchang University. In brief, 4-week nude mice were purchased from Shanghai Slac Laboratory Animal Company (Shanghai, China) and the flanks of mice were injected with 1 × 10^7^ stably infected SK-N-SH cells, including shRNA-circDGKB, Lentiv-circDGKB, shRNA-NC, Lentiv-NC, mimic-NC, mimic-miR-873, inhibitor-miR-873, inhibitor-NC, Lentiv-GLI1, shRNA-GLI1. After 4 weeks, mice were euthanized and tumor weights were measured.

### Statistical Analysis

Date from three independent experiments are shown as mean ± standard deviation (SD). Comparison among 2 groups or > 2 groups was executed by using the Student's *t*-test or one-way analysis of variance (ANOVA) using SPSS 17.0 software. *P* < 0.05 was considered as statistically significant. ROC (receiver operating characteristic) curve was established to evaluate clinical diagnostic value of circDGKB in NB, with larger area under the curve meaning the higher prognostic. The relationship between circDGKB expression levels and the overall survival rates of NB patients was assessed by using Kaplan-Meier analysis with log rank test.

## Result

### circDGKB Was Highly Expressed in NB Tissue Specimens

**Figure 1:**
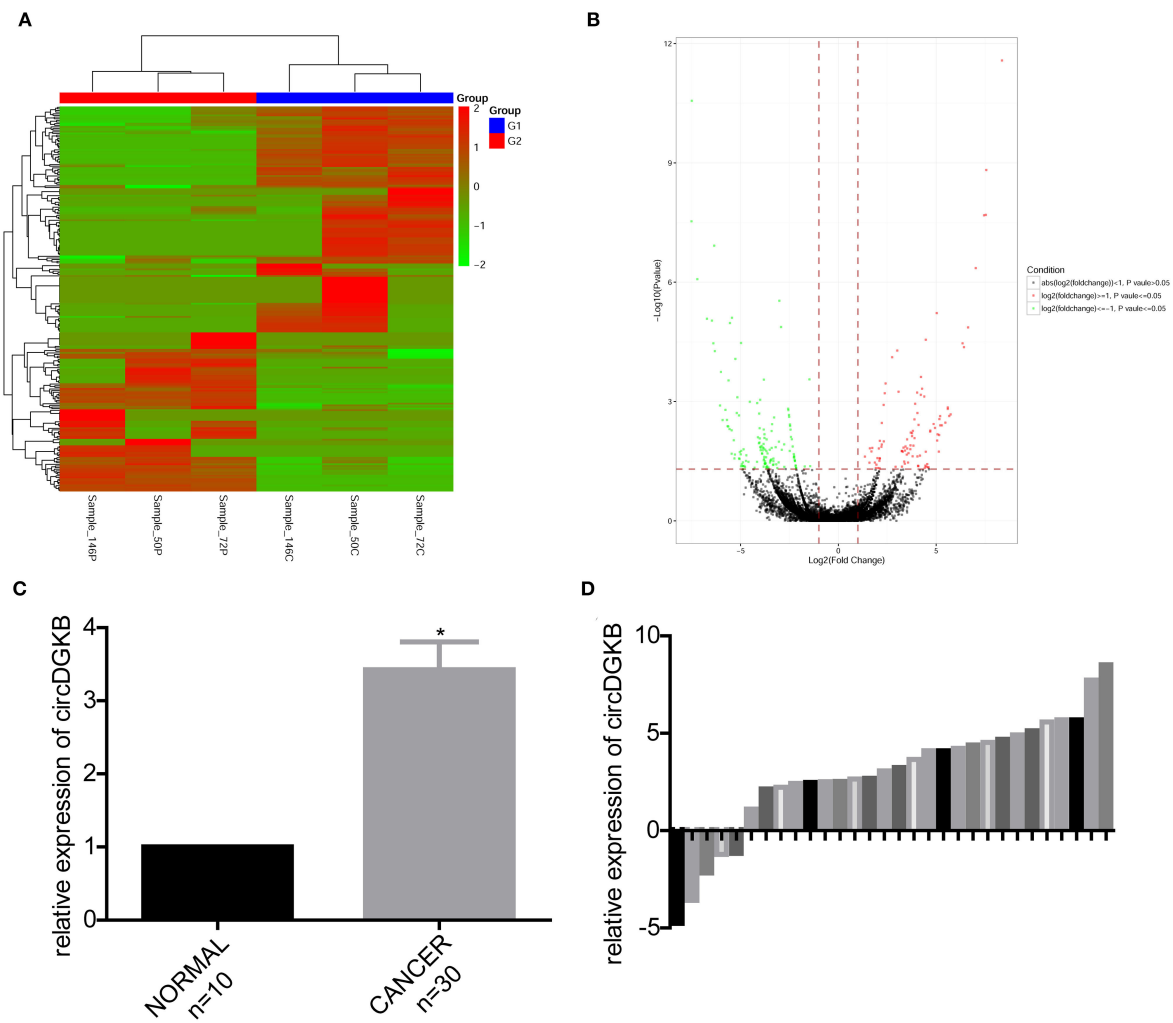
CircDGKB was overexpressed in NB specimens. **(A)** Heat map of genes in 3 NB tissue specimens and 3 human normal dorsal root ganglia. Red represented the upregulated genes while green were the downregulated genes. **(B)** Volcano plot of genes in 3 NB tissues and 3 human normal dorsal root ganglia. Red represented the upregulated genes while green were the downregulated genes. **(C)** QRT-PCR was taken to evaluate the expression of circDGKB in 30 NB tissue specimens and 10 human normal dorsal root ganglia, *P < 0.05. **(D)** QRT-PCR was taken to detect the relative expression levels of circDGKB in 30 NB tissue specimens to 10 normal dorsal root ganglia.

To find the specific circRNAs which are differentially expressed in NB specimens, 3 NB tissues obtained from NB patients and 3 normal dorsal root ganglia collected from interrupted pregnancies were subjected to circRNA microarray assay. A total of 235 circRNAs were found to be differentially expressed in NB specimens compared with the dorsal root ganglia, among which 138 circRNAs were upregulated and 97 circRNAs were downregulated (Figures 1A,B). And circDGKB expression was increased, highest among the 138 upregulated circRNAs. The qRT-PCR assay was carried out to confirm the expression pattern of circDGKB in 30 paired NB tissues and 10 normal dorsal root ganglia. The results showed that the expression level of circDGKB in NB tissues was significantly increased (Figure 1C). Figure 1D demonstrated the relative expression levels of circDGKB in 30 NB tissues to normal dorsal root ganglia. Taken together, these results demonstrated that circDGKB was overexpressed in NB tissues.

### circDGKB Accelerated the Progression of NB

**Figure 2:**
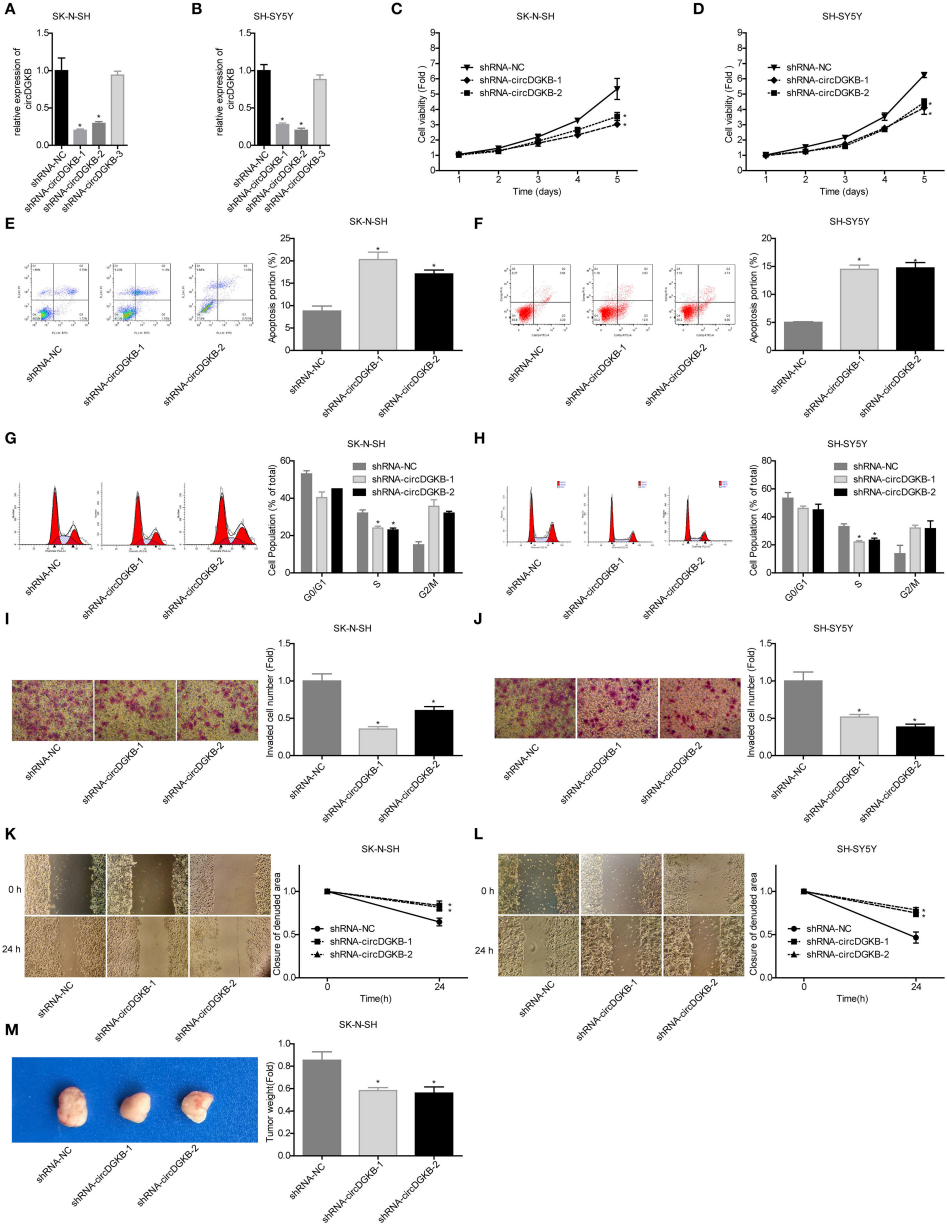
Evaluation of the roles of circDGKB knockdown in NB cell proliferation, apoptosis and cell cycle, migration, invasion, and tumorigenesis. **(A,B)** QRT-PCR was taken to detect the infected efficiencies of shRNAs-circDGKB in SK-N-SH and SH-SY5Y cell lines. **(C,D)** CCK-8 assay was used to detect the proliferation of SK-N-SH and SH-SY5Y cells. **(E,F)** Flow cytometry was adopted to detected cell apoptosis. **(G,H)** Flow cytometry was used to determine the distribution of cell cycle. **(I,J)** Transwell experiment was performed to evaluate the invasion of SK-N-SH and SH-SY5Y cells. **(K,L)** Wound healing assay was performed to detect the migration of SK-N-SH and SH-SY5Y cells. **(M)** Nude mice tumorigenicity experiments was adopted to test the tumorigenesis of SK-N-SH cells *in vivo* (*P < 0.05 vs. shRNA-NC group).

To investigate the role of circDGKB in the progression of NB, gain of function and loss of function experiments were conducted in NB cell lines SK-N-SH and SH-SY5Y. QRT-PCR assay was taken to test the infection efficiencies of shRNA-circDGKB and Lentiv-circDGKB. Compared with the control group, circDGKB expression was obviously reduced after the cells were infected with shRNA-circDGKB in both SK-N-SH and SH-SY5Y cell lines (Figures 2A,B). CCK-8 assay demonstrated that downregulation of circDGKB prominently attenuated cell proliferation in SK-N-SH and SH-SY5Y cells (Figures 2C,D). Flow cytometry showed that circDGKB downregulation increased cell apoptosis (Figures 2E,F). In addition, we found that circDGKB downregulation could lead to S phase reduction (Figures 2G,H). Moreover, the wound healing assay and transwell invasion assay were performed to examine the migration and invasion of SK-N-SH and SH-SY5Y cells. As shown in Figures 2I–L, downregulation of circDGKB significantly impaired cell invasion and migration abilities in SK-N-SH and SH-SY5Y cells. Furthermore, the *in vivo* assay was carried out to explore the function of circDGKB in tumor formation. The result showed that downregulation of circDGKB reduced tumor formation in SK-N-SH cells (Figure 2M).

**Figure 3:**
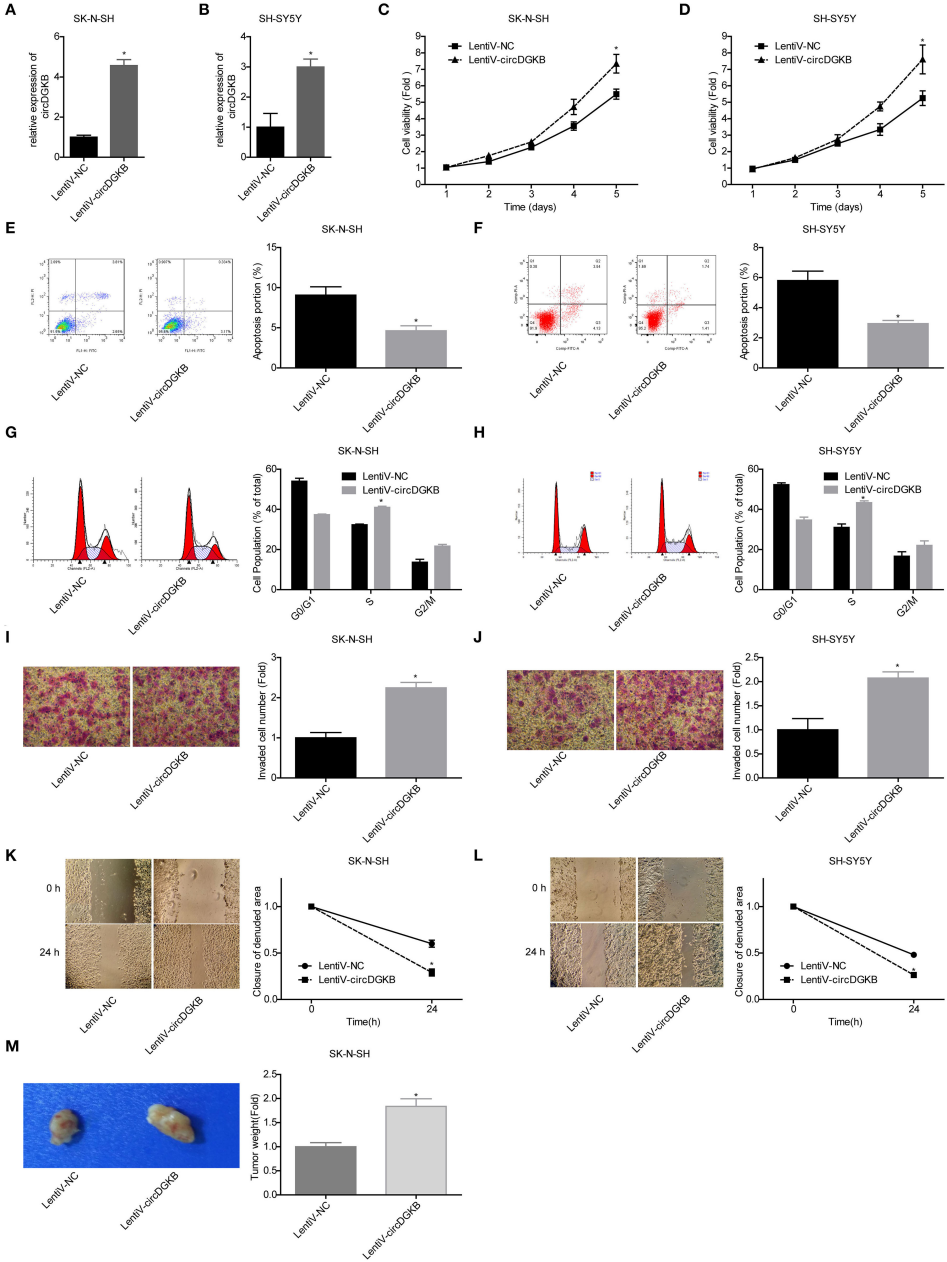
Evaluation of the roles of circDGKB overexpression in NB cell proliferation, apoptosis and cell cycle, migration, invasion, and tumorigenesis. **(A,B)** QRT-PCR was taken to detect the infected efficiencies of Lentiv-circDGKB in SK-N-SH and SH-SY5Y cell lines. **(C,D)** CCK-8 assay was used to detect the proliferation of SK-N-SH and SH-SY5Y cells. **(E,F)** Flow cytometry was adopted to detected cell apoptosis. **(G,H)** Flow cytometry was used to determine the distribution of cell cycle. **(I,J)** Transwell experiment was performed to evaluate the invasion of SK-N-SH and SH-SY5Y cells. **(K,L)** Wound healing assay was performed to detect the migration of SK-N-SH and SH-SY5Y cells. **(M)** Nude mice tumorigenicity experiments was adopted to test the tumorigenesis of SK-N-SH cells *in vivo* (*P < 0.05 vs. Lentiv-NC group).

To the contrary of shRNA-circDGKB infection, circDGKB expression was markedly increased when the cells were infected with Lentiv-circDGKB in both SK-N-SH and SH-SY5Y cell lines (Figures 3A,B). CCK-8 assay demonstrated that overexpression of circDGKB obviously enhanced the proliferative capacity of SK-N-SH and SH-SY5Y cells (Figures 3C,D). Flow cytometry showed that circDGKB overexpression significantly inhibited cell apoptosis (Figures 3E,F). In addition, we found that overexpression of circDGKB could induce cell S phase arrest (Figures 3G,H). Moreover, the wound healing assay and transwell invasion assay showed that overexpression of circDGKB significantly promoted cell invasion and migration in SK-N-SH and SH-SY5Y cells (Figures 3I–L). Furthermore, the *in vivo* assay showed that overexpression of circDGKB obviously promoted the tumorigenesis of SK-N-SH cells (Figure 3M). These results showed that circDGKB could accelerate the progression of NB.

### miR-873/GL1-3 Axis Was a Target of circDGKB in NB Cells

**Figure 4:**
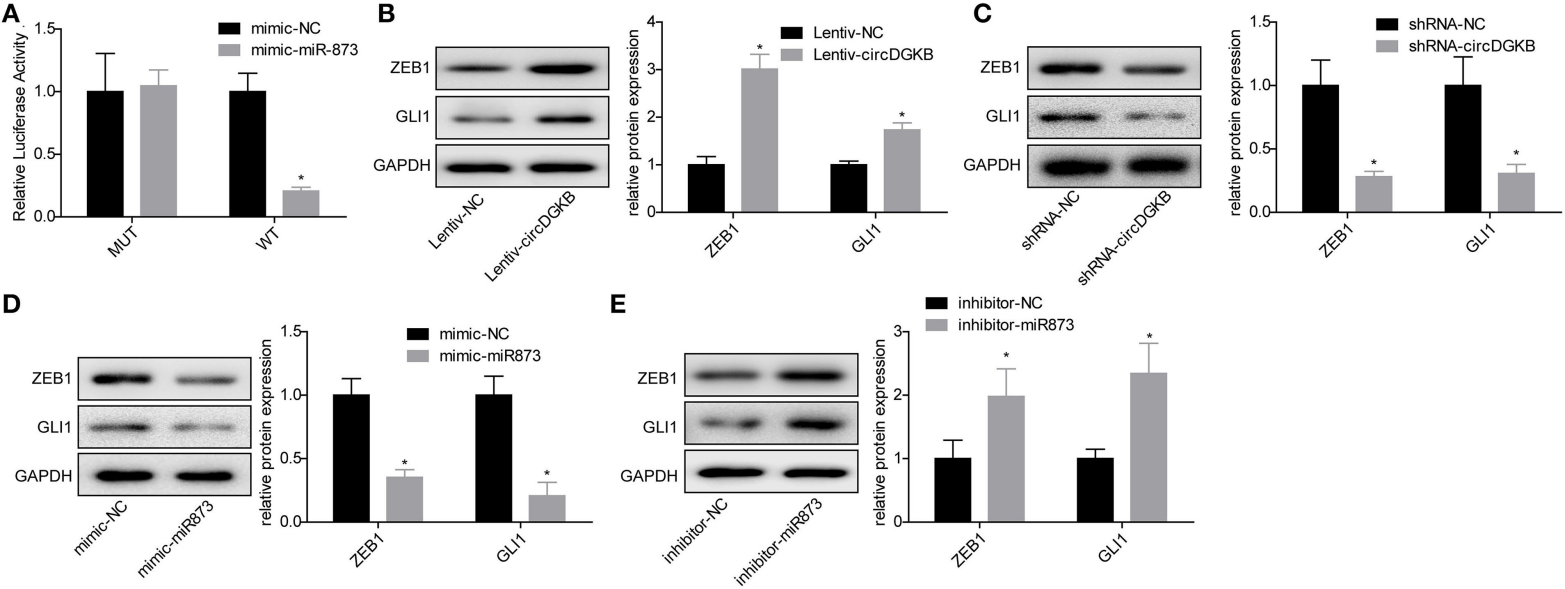
circDGKB targeted miR-873/GLI1 axis in NB SK-N-SH cells. **(A)** Luciferase reporter gene assay was adopted to confirm the interaction between circDGKB and miR-873. **(B)** Western blot was taken to calculate the relative protein levels of ZEB1 and GLI1 in SK-N-SH cells after circDGKB overexpression, *P < 0.05. **(C)** Relative protein levels of ZEB1 and GLI1 in SK-N-SH cells were detected following circDGKB downregulation, *P < 0.05. **(D)** Relative protein levels of ZEB1 and GLI1 in SK-N-SH cells were calculated after miR-873 was upregulated, *P < 0.05. **(E)** Relative protein levels of ZEB1 and GLI1 in SK-N-SH cells were examined by western blot after miR-873 was downregulated, *P < 0.05.

Previous studies proved that circRNA could act as a sponge of miRNA. To explore the underlying targets of circDGKB, we performed miRNA prediction with miRanda. As shown in Figure 4A, results of the luciferase reporter assay showed that miR-873 overexpression dramatically reduced the luciferase activity of circDGKB-WT, whereas the mutation of binding sites abolished this effect. The further bioinformatics analysis predicted that GLI1 was a target for miR-873. To verify the foreclosed results, we conducted “gain” and “lost” experiment in SK-N-SH cells. The results of western blot indicated that circDGKB increased the expression of GLI1 and ZEB1 under the regulation of GLI1 (16) (Figure 4B), while circDGKB downregulation reduced GLI1 and ZEB1 expression (Figure 4C). In addition, miR-873 overexpression downregulated GLI1 and ZEB1 expression (Figure 4D), while knockdown of miR-837 upregulated the expression of ZEB1 and GLI1 (Figure 4E). These results suggested that circDGKB increased the expression of ZEB1 and GLI1 via targeting miR-873.

### miR-873 Played a Vital Role in the Progression of NB

**Figure 5:**
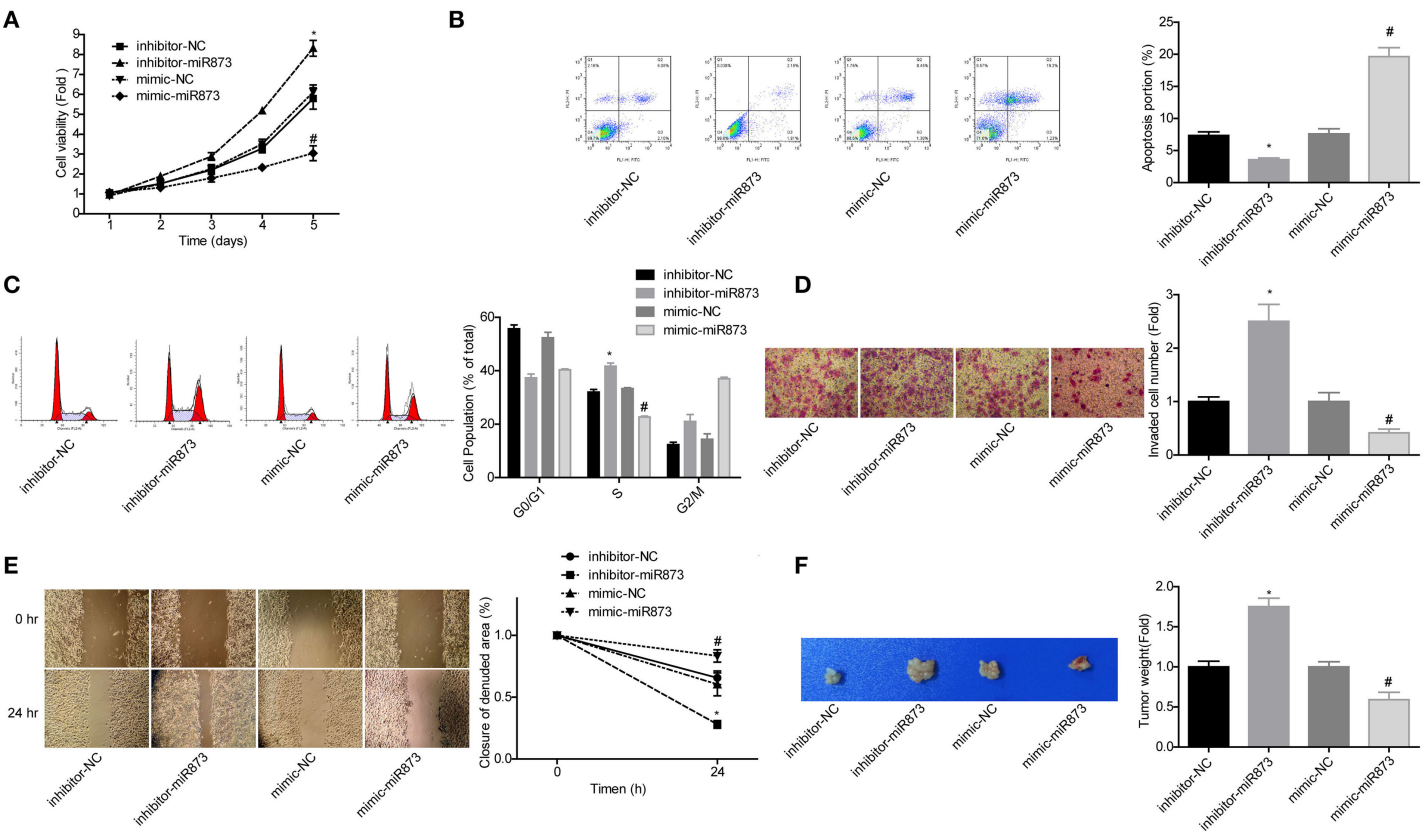
MiR-873 played critical roles in the progression of NB. **(A)** Proliferation of SK-N-SH cells was detected by CCK-8 assay. **(B)** Apoptosis of SK-N-SH cells was detected by flow cytometry. **(C)** Cell cycle of SK-N-SH cells was assessed by flow cytometry. **(D)** Cell invasion and **(E)** migration were determined by transwell experiment and wound healing assay in SK-N-SH cells. **(F)** Xenograft model was established to detect the tumorigenesis of SK-N-SH cells *in vivo* (*P < 0.05, vs. inhibitor-NC group, ^#^*P* < 0.05, vs. mimic-NC group).

To explore the role of miR-873 in the progression of NB, the gain of function and loss of function experiments were carried out in SK-N-SH cells. MiR-873 overexpression significantly reduced the proliferation of SK-N-SH cells, while downregulation led to the opposite results (Figure 5A). Cell apoptosis and S phase arrest were induced by miR-873 upregulation in SK-N-SH cells, and knockdown of miR-873 caused the opposite effects (Figures 5B,C). The results of transwell experiments and wound healing assay showed that overexpression of miR-873 promoted the invasion and migration of SK-N-SH cells, and vice versa (Figures 5D,E). In addition, the *in vivo* assay demonstrated that overexpression of miR-873 inhibited the tumor formation ability of SK-N-SH cells, and vice versa (Figure 5F). These results suggested that miR-873 inhibited the progression of NB.

### circDGKB Promoted NB Progression via Targeting miR-873

**Figure 6:**
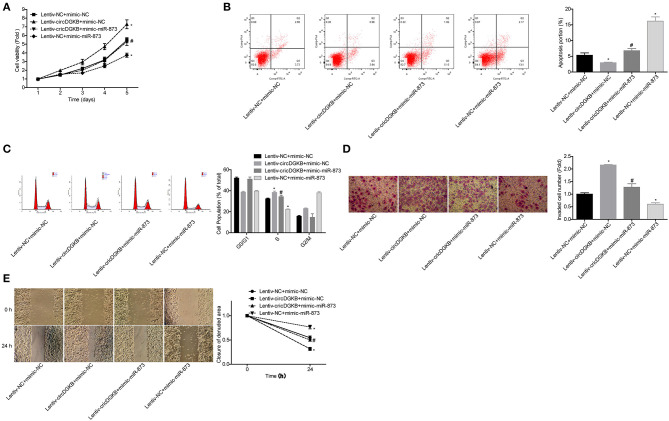
circDGKB promoted NB progression by targeting miR-873. **(A)** Proliferation of SK-N-SH cells was detected by CCK-8 assay. **(B)** Apoptosis of SK-N-SH cells was detected by flow cytometry. **(C)** Cell cycle of SK-N-SH cells was assessed by flow cytometry. **(D)** Cell invasion and **(E)** migration were determined by transwell experiment and wound healing assay in SK-N-SH cells. (*P < 0.05, vs. Lentiv-NC+mimic-NC group, ^#^*P* < 0.05, vs. Lentiv-circDGKB+mimic-NC group).

Then, we explored whether miR-873 was involved in circDGKB-mediated NB progression. The results showed that miR-873 overexpression significantly weakened circDGKB roles in promoting cell proliferation (Figure 6A), S phase arrest (Figure 6C), invasion (Figure 6D), and migration (Figure 6E), as well as inhibiting cell apoptosis (Figure 6B). These results demonstrated that circDGKB promoted NB progression via targeting miR-873.

### miR-873 Promoted NB Progression via Targeting GLI1

**Figure 7:**
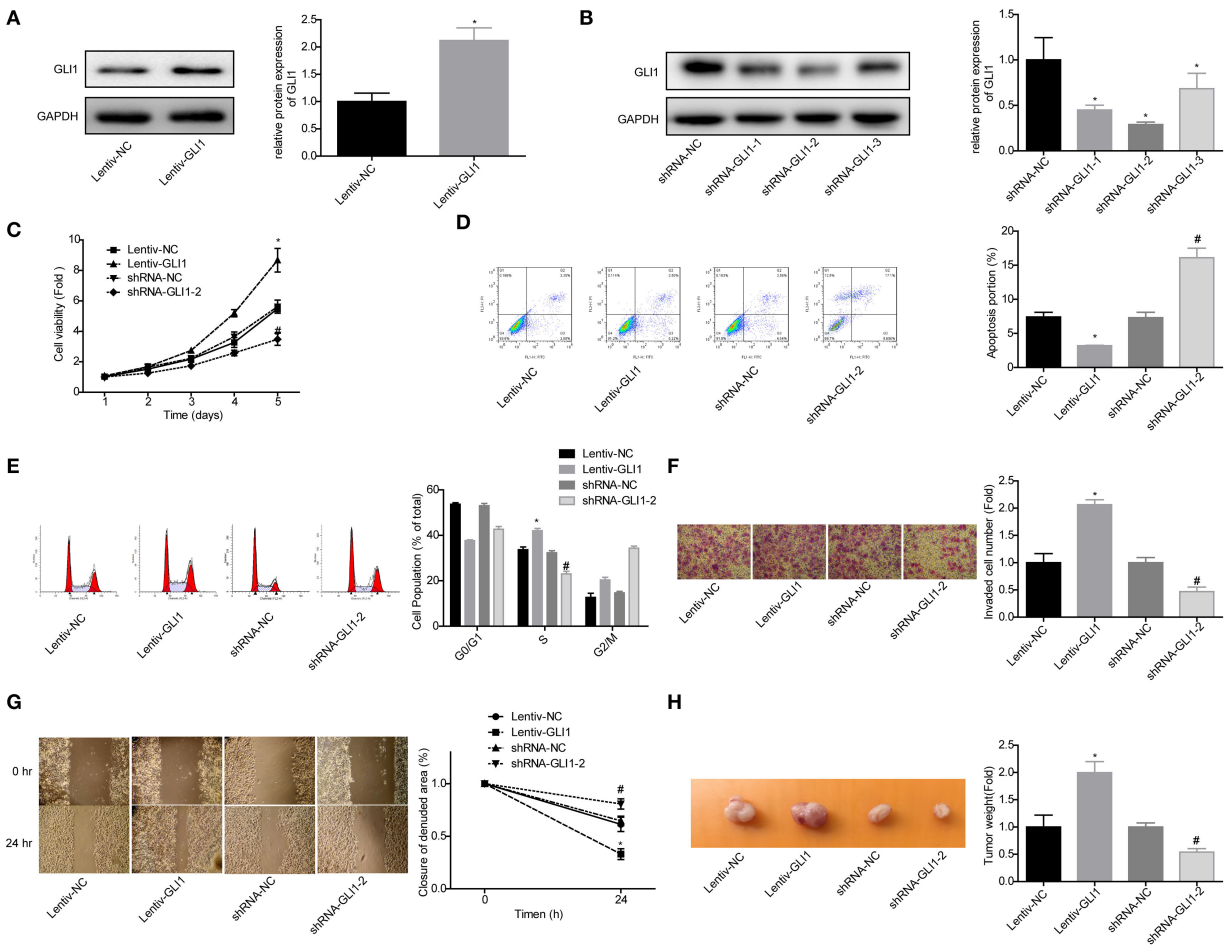
GLI1 served as an oncogene in NB progression. **(A,B)** Western blot was taken to detect the infected efficiencies of Lentiv-GLI1 and shRNAs-GLI-shRNAs in SK-N-SH cells, *P < 0.05. **(C)** CCK-8 assay was taken to detect the proliferation of SK-N-SH cells. Flow cytometry assay was adopted to detect **(D)** cell apoptosis and **(E)** cell cycle. **(F)** Cell invasion and **(G)** migration in SK-N-SH cells were detected by using the transwell chambers and wound healing assay. **(H)** Xenograft model was established to detect the tumorigenesis of SK-N-SH cells. (*P < 0.05, vs. Lentiv-NC group, ^#^*P* < 0.05, vs. shRNA-NC group).

Next, we explored GLI1 role in the progression of NB in SK-N-SH cells. Western blot assay was used to verify the efficiency of lentiv-GLI1 and shRNA-GLI1 (Figures 7A,B). As expected, overexpression of GLI1 obviously promoted the proliferation of SK-N-SH cells, while downregulation of GLI1 caused the opposite effect (Figure 7C). GLI1 upregulation significantly inhibited apoptosis and induced S phase arrest in SK-N-SH cells, and vice versa (Figures 7D,E), as well as promoted cell invasion, migration, and tumorigenesis *in vivo*, and vice versa (Figures 7F–H). These results indicated that GLI1 accelerated the progression of NB.

**Figure 8:**
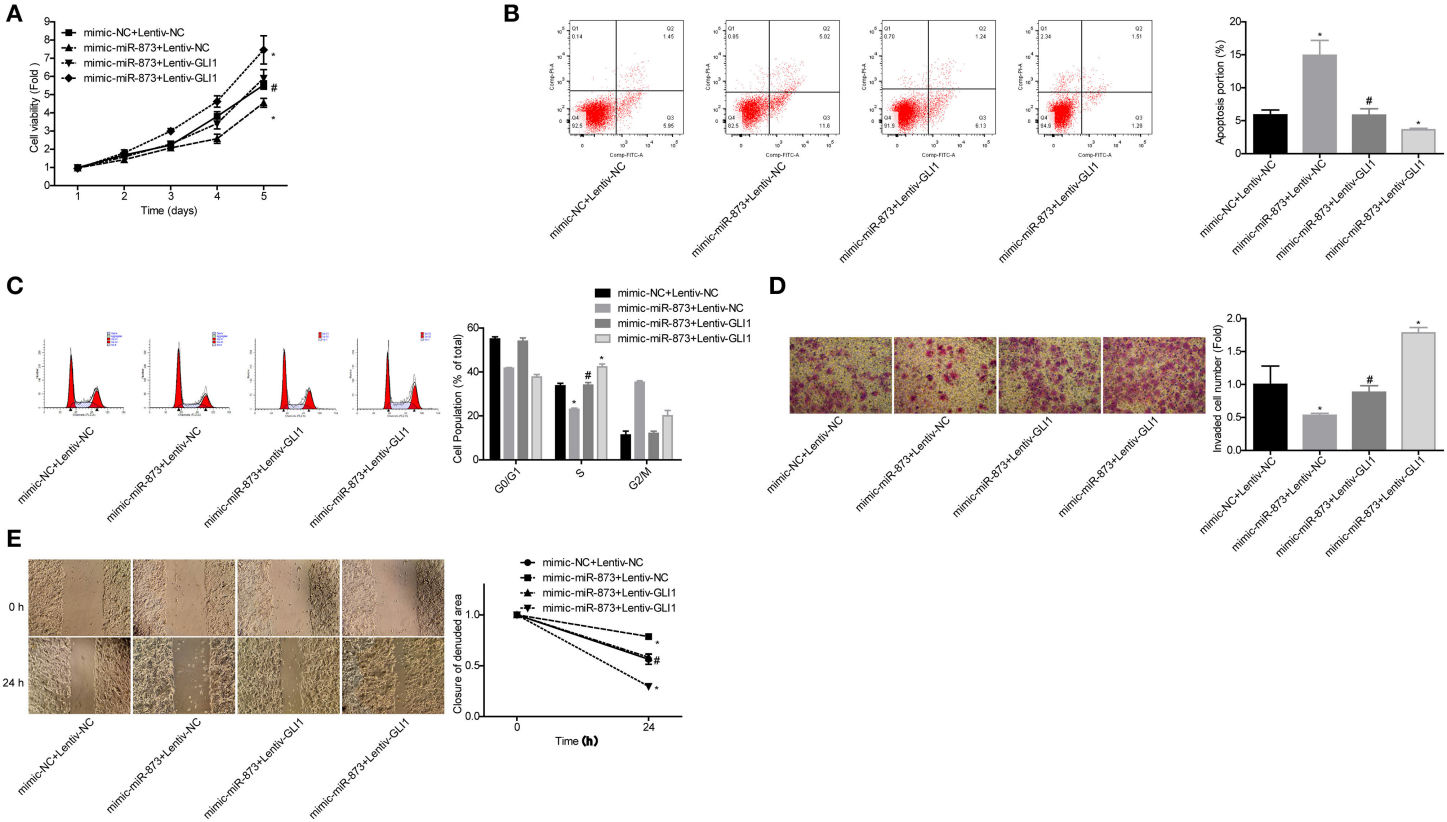
miR-873 inhibited NB progression by targeting GLI1. **(A)** Proliferation of SK-N-SH cells was detected by CCK-8 assay. **(B)** Apoptosis of SK-N-SH cells was detected by flow cytometry. **(C)** Cell cycle of SK-N-SH cells was assessed by flow cytometry. **(D)** Cell invasion and **(E)** migration were determined by transwell experiment and wound healing assay in SK-N-SH cells. (*P < 0.05, vs. mimic-NC+Lentiv-NC group, ^#^*P* < 0.05, vs. mimic-miR-873+Lentiv-NC group).

To explore whether GLI1 was implicated in miR-873-mediated NB progression inhibition, the rescued assays were carried out. Compared with the mimic-miR-873+lentive-NC group, cell proliferation (Figure 8A), invasion (Figure 8D), and migration (Figure 8E) abilities were significantly enhanced, while cell apoptosis were reduced (Figure 8B) and cell numbers in S phase were increased (Figure 8C) in mimic-miR-873+lentiv-GLI1 group. These findings suggested that miR-873 promoted NB progression via targeting GLI1.

### circDGKB Acted as a Potential Marker for NB Diagnosis

**Figure 9:**
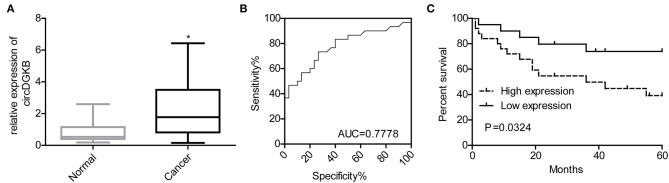
circDGKB showed potential in the diagnosis of NB. **(A)** Expression level of circDGKB in blood samples of NB patients was detected by qRT-PCR, *P < 0.05. **(B)** ROC curve was established to evaluate the clinical value of circDGKB in the diagnosis of NB. **(C)** The effect of circDGKB expression level in predicting the overall survival rates of NB patients was assessed by using Kaplan-Meier analysis with log rank test (*n* = 45, *P* = 0.034).

To investigate whether circDGKB could act as a diagnostic marker for NB, we detected the expression of circDGKB in the blood samples of NB patients and healthy subjects. The results of qRT-PCR showed that circDGKB expression in blood was prominently upregulated in NB patients compared with normal subjects (Figure 9A). The results of ROC analyses demonstrated that the level of circDGKB had an important clinical value in the diagnosis of NB with an AUC (area under the curve) of 0.7778 (Figure 9B). The results of Kaplan-Meier analysis showed that patients with high level of circDGKB had lower survival rates (Figure 9C).

## Discussion

circRNA produced from exons or introns is stable existing and was first found in RNA viruses in the 1970s (17, 18), at which time the circRNA was thought to have no biological function (19–23). To date, a great deal of studies proved that circRNAs were ubiquitous in mammalian cells and played critical roles in regulating gene expression by targeting miRNA or other molecules including linear slicing and proteins (24–26). Growing evidences have proved that circRNAs play a key role in many diseases, including systemic lupus erythematous (27), atherosclerosis (28), Neuropsychiatric Disorders (29), Alzheimer's disease (30), cardiovascular diseases (31), and cancers (32). Qiu et al. (33) identified that the expression level of circ000911 could promote the proliferation and invasion of non-small-cell lung cancer (NSCLC) cells by interacting with miR-22-3p directly and then enhanced the expression level of galectin-1 (Gal-1), p-AKT, and p-ERK1/2. Fang et al. (34) indicated that circ100290 was highly expressed in colorectal cancer (CRC) tissues and cell lines; silence of circ100290 inhibited the proliferation, migration, and invasion of CRC cells, but promoted cell apoptosis. In addition, Fang et al. (34) also proved that circ100290 expression level showed positive correlation with CRC metastasis and negative correlation with patients' prognosis. Gao et al. (35) illustrated that circ0006528 promoted the proliferation, migration, and invasion of breast cancer cells by targeting miR-7-5p/raf1/MAPK/ERK pathways. In the present study, we found that circDGKB expression was prominently unregulated in NB tissues; overexpression of circDGKB promoted the proliferation, migration, and invasion of SK-N-SH cells, and inhibited cell apoptosis and induced S phase arrest, while circDGKB downregulation showed the reverse effects.

MiRNAs, a class of non-coding RNA with ~22 nucleotides, have been proved to play a vital role in tumorigenesis by regulating the expression of oncogenes or tumor suppressive genes. For instance, miR-776 was downregulated in colon cancer, and low expression level of miR-776 was associated with low survival rate of patients. In addition, the authors proved that overexpression of miR-776 obviously inhibited cell proliferation and initiated cell apoptosis in colon cancer cells by targeting MDM4/p53 pathway (36). miR-20a-5p was identified to be highly expressed in triple-negative breast cancer (TNBC) tissues, which promoted TNBC progression by targeting Runt-related transcription factor 3 (RUNX3) (37). In our work, we proved that miR-873 was a direct target of circDGKB, and inhibition of the expression of miR-873 enhanced the proliferation, migration, invasion, and S phase arrest of SK-N-SH cells and reduced cell apoptosis. This results were consistent with previous studies, which indicated the suppressive role of miR-873 in cancers including CRC (38, 39), breast cancer (40), NSCLC (41), and gastric cancer (42). Furthermore, we found that miR-873 overexpression significantly rescued circDGKB role in promoting NB progression, illustrating that circDGKB promoted NB progression by targeting miR-873.

It's well-known that Hedgehog (HH) signaling pathway plays critical roles in amount of cancers, including NB (43–46). GLI1, a terminal effectors of the HH factors, was reported to serve as an oncogene in wide ranges of cancers, such as breast cancer (47), CRC (48), prostate cancer (49), glioma (50), pancreatic cancer (51), gastric cancer (52), and cervical cancer (53). Noticeably, GLI1 was reported to be a direct target of miR-873-5p in gastric cancer (42). Similarly, in our work, GLI1 was also demonstrated as a direct target of miR-873 in NB cells, and GLI1 overexpression could markedly weaken miR-873 roles in inhibiting cell proliferation, migration, and invasion of SK-N-SH cells and inducing cell apoptosis and S phase arrest, suggesting that miR-873 inhibited NB progression via targeting GLI1.

Collectively, our work demonstrated that circDGKB acted as an oncogene in NB progression by targeting miR-873/GLI1 axis. In addition, we found the high expression level of circDGKB was associated with the poor prognosis of NB patients, suggesting that circDGKB could be used as a diagnostic marker for NB. Our study suggested that circDGKB was a potential therapeutic target and diagnostic marker for NB.

## Data Availability Statement

The datasets generated for this study can be found in the National Center for Biotechnology Information. Circular RNA DGKB promotes the progression of neuroblastoma by targeting miR-873-GLI1 axis:PRJNA554935; Human sample from Homo sapiens:SAMN12287376, SAMN12287375, SAMN12287374, SAMN12287373, SAMN12287372, and SAMN12287371; circRNA seq of neuroblastma:SRR9694951, SRR9694952, SRR9694948, SRR9694947, SRR9694949, and SRR9694950.

## Ethics Statement

The studies involving human participants were reviewed and approved by the Research Ethics Committee of Second Affiliated Hospital of Nanchang University. Written informed consent to participate in this study was provided by the participants' legal guardian/next of kin. The animal study was reviewed and approved by the Research Ethics Committee of Second Affiliated Hospital of Nanchang University.

## Author Contributions

SZ and XY came up with the conception and design of study. JYang and LY performed the experiment and acquired the data. JYan, YX, and WL analyzed the data. JX, JL, and DX performed some operations such as specimen acquisition and disposal. JYang drafted the manuscript. SZ and XY revised the manuscript critically for important intellectual content. All authors agreed to publish the manuscript.

## Conflict of Interest

The authors declare that the research was conducted in the absence of any commercial or financial relationships that could be construed as a potential conflict of interest.
