# Supplementary figures and images for: Circular RNA DGKB Promotes the Progression of Neuroblastoma by Targeting miR-873/GLI1 Axis
Source: Front Oncol. 2020 Jul 23;10:1104. doi: 10.3389/fonc.2020.01104 (PMC7390925; doi:10.3389/fonc.2020.01104)

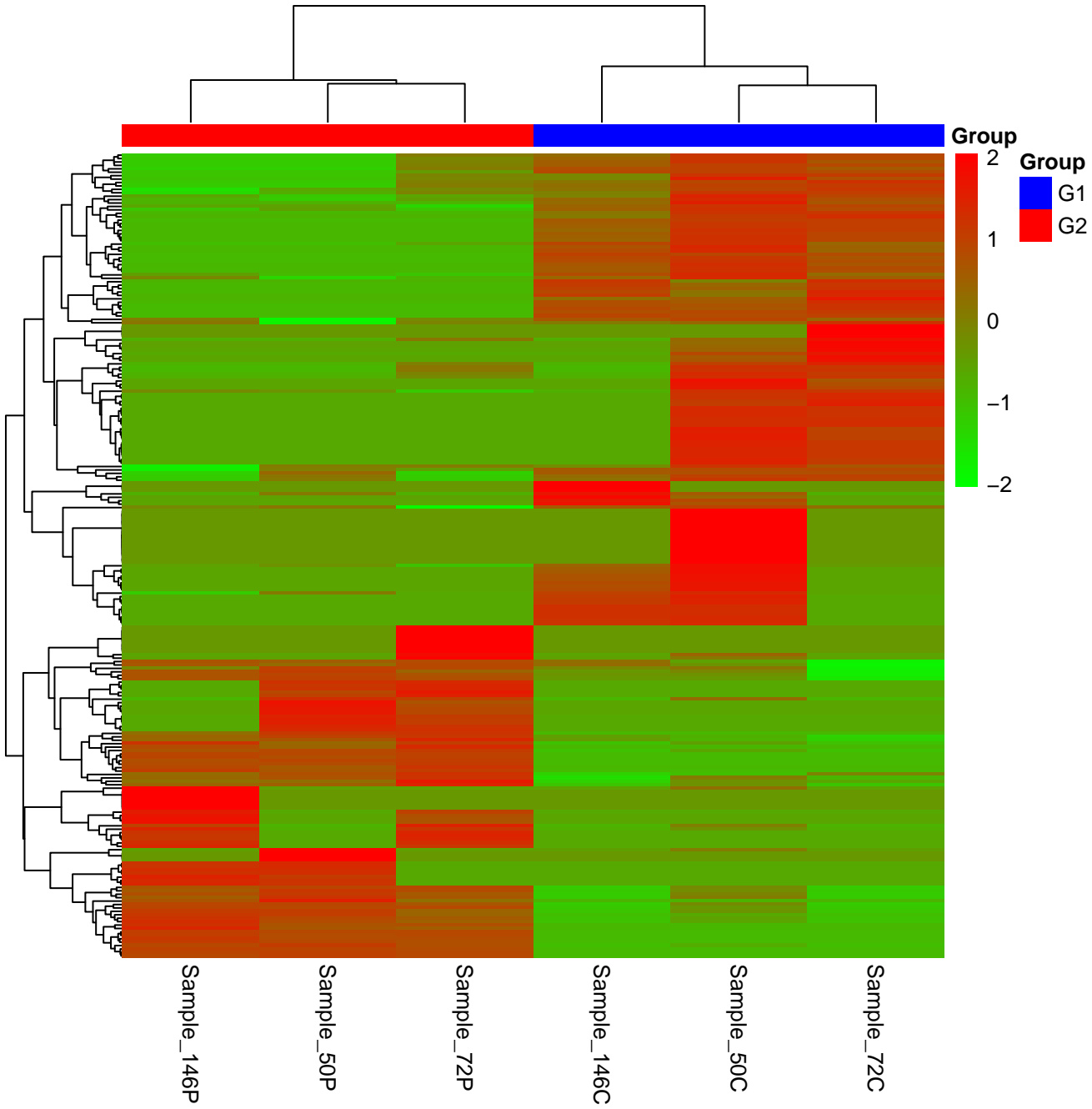

Supplement: Supplementary file 1 [file Data_Sheet_1.ZIP › Supplementary file/P_vs_C/diff_cirRNA_exp_for_heatmap.pdf]

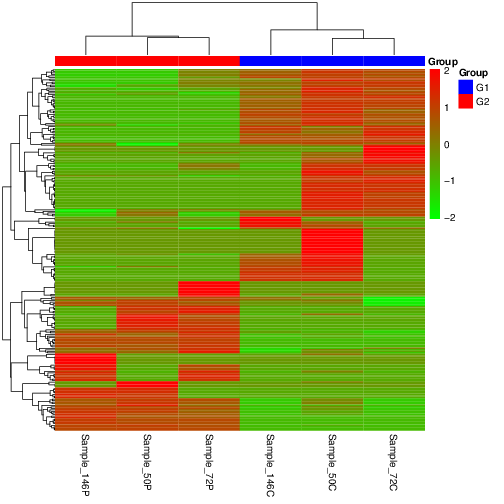

Supplement: Supplementary file 1 [file Data_Sheet_1.ZIP › Supplementary file/P_vs_C/diff_cirRNA_exp_for_heatmap.png]

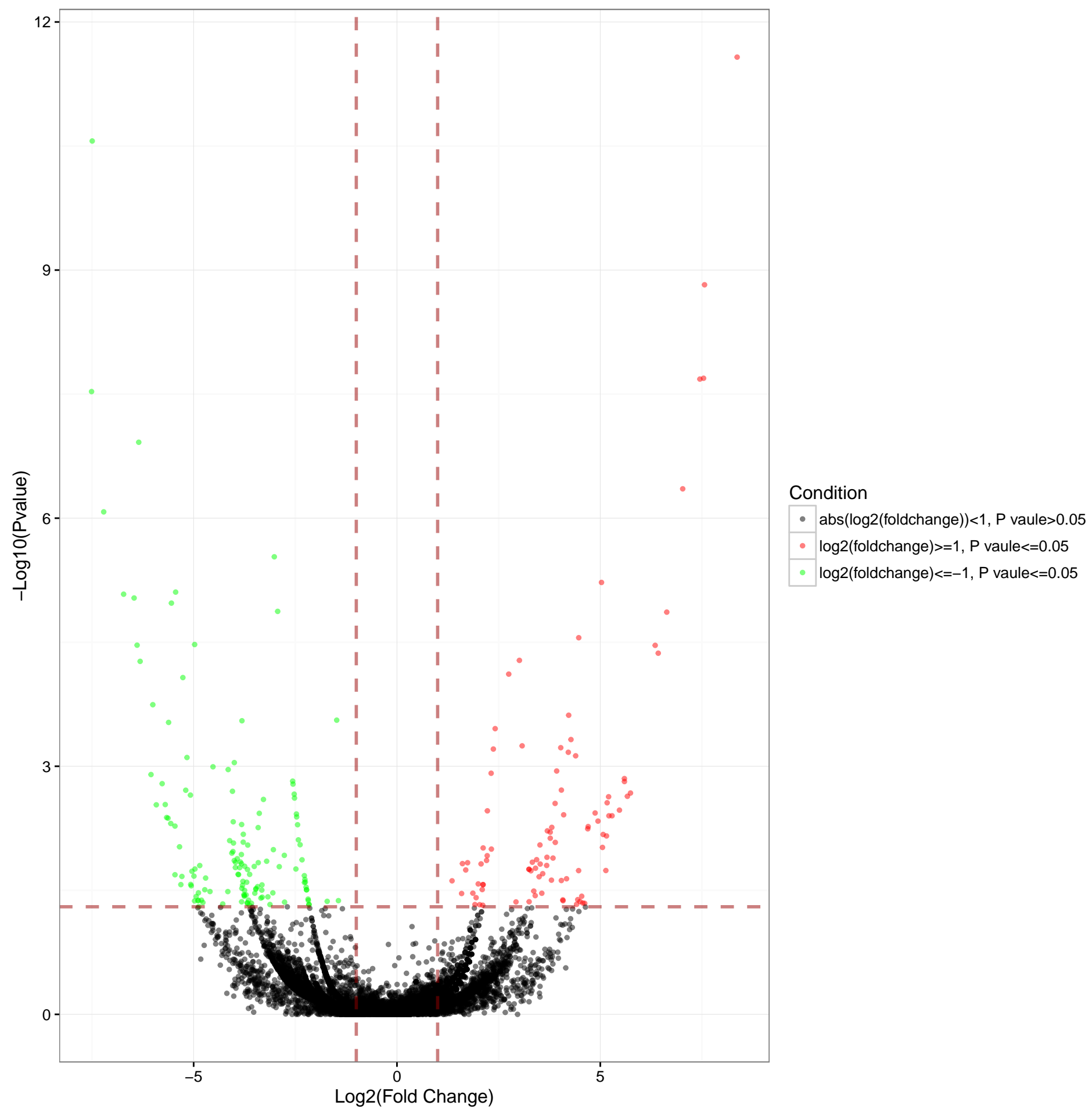

Supplement: Supplementary file 1 [file Data_Sheet_1.ZIP › Supplementary file/P_vs_C/volcano_plot.pdf]

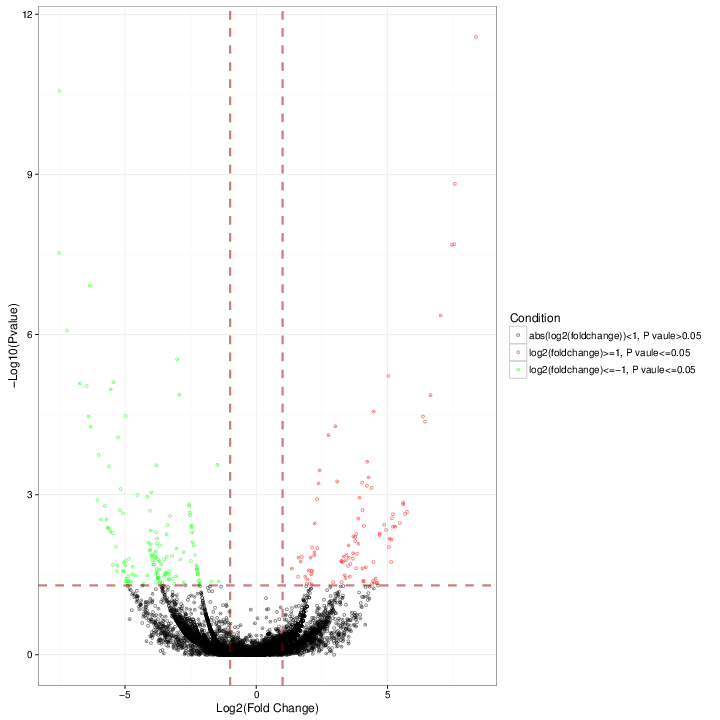

Supplement: Supplementary file 1 [file Data_Sheet_1.ZIP › Supplementary file/P_vs_C/volcano_plot.png]

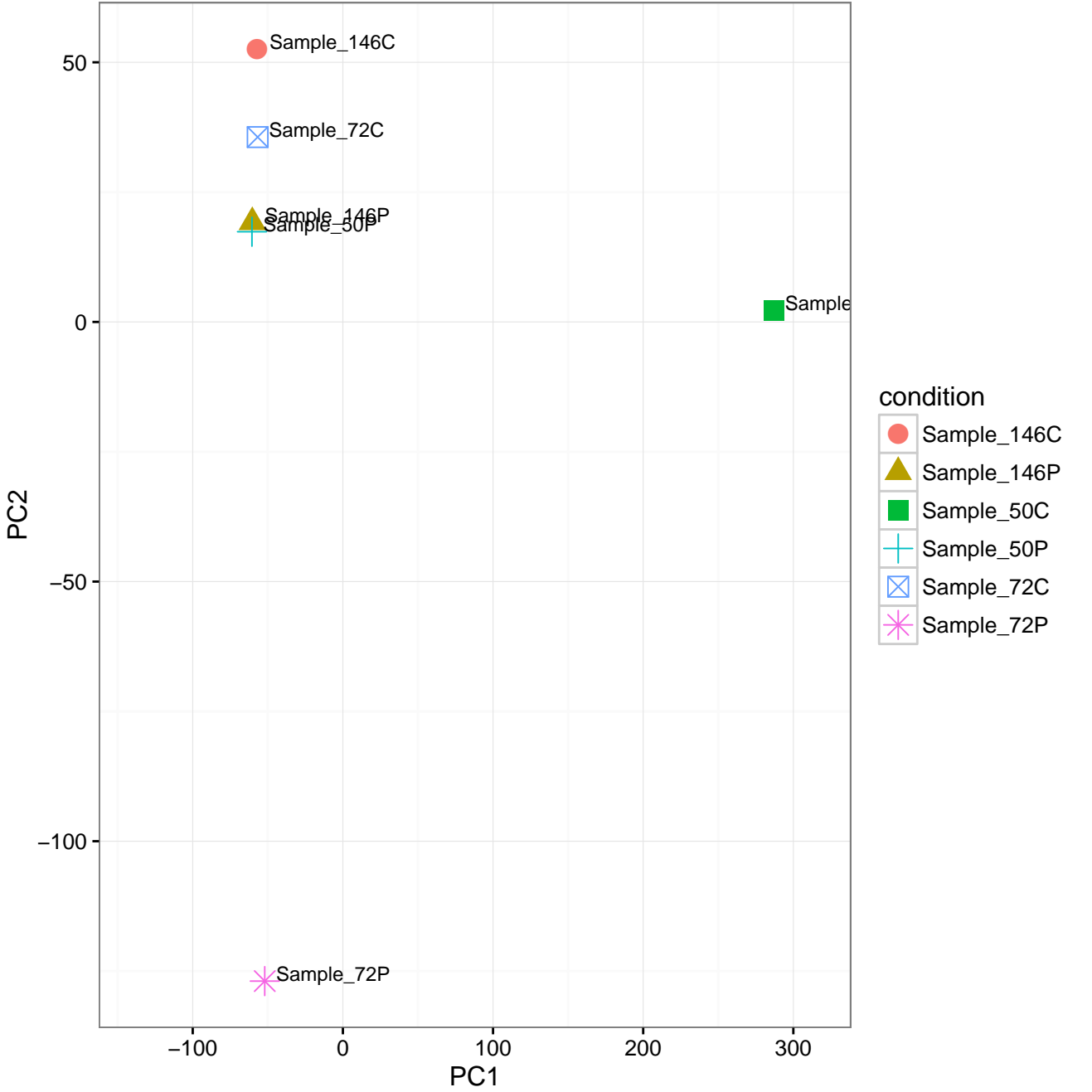

Supplement: Supplementary file 1 [file Data_Sheet_1.ZIP › Supplementary file/sample_cir_BSRP_PCA.pdf]

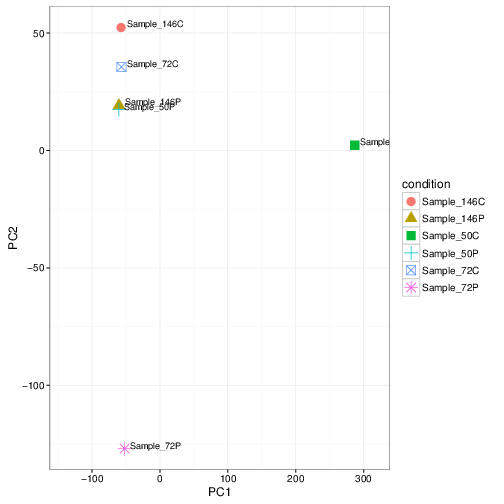

Supplement: Supplementary file 1 [file Data_Sheet_1.ZIP › Supplementary file/sample_cir_BSRP_PCA.png]

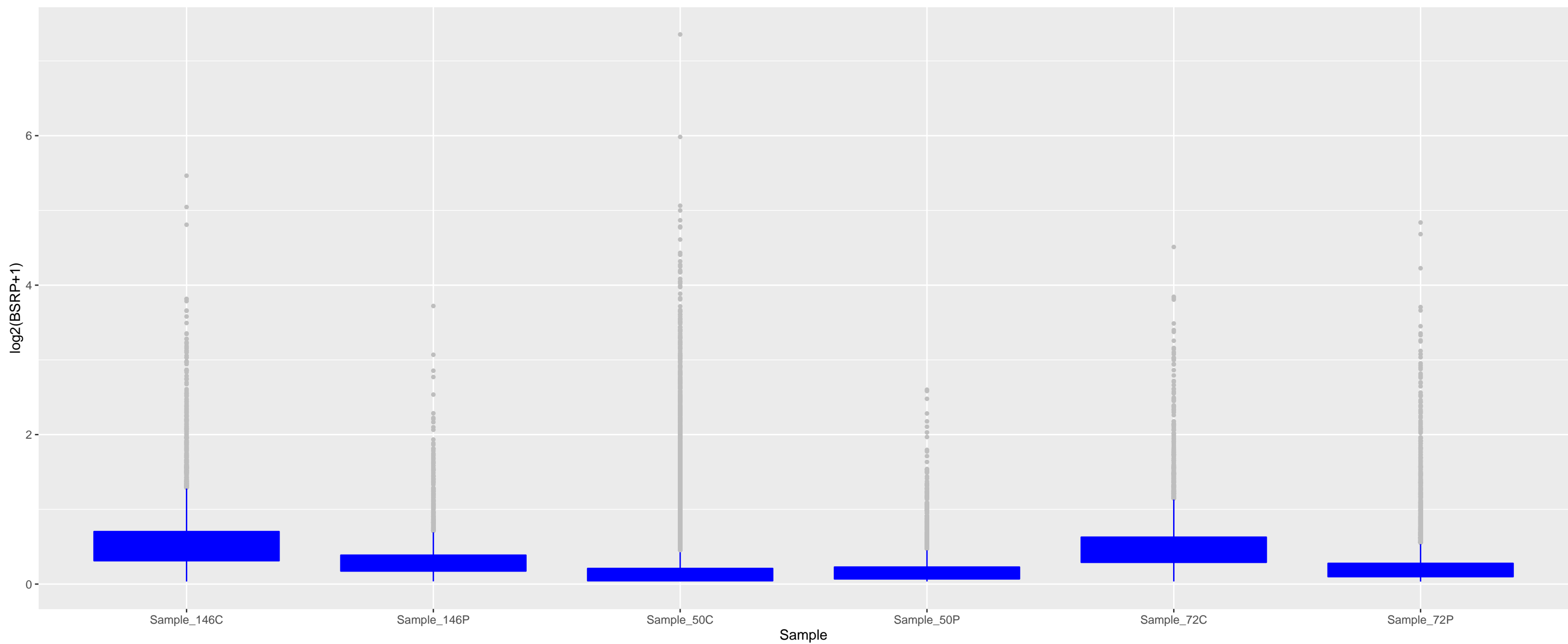

Supplement: Supplementary file 1 [file Data_Sheet_1.ZIP › Supplementary file/sample_cir_BSRP_boxplot.pdf]

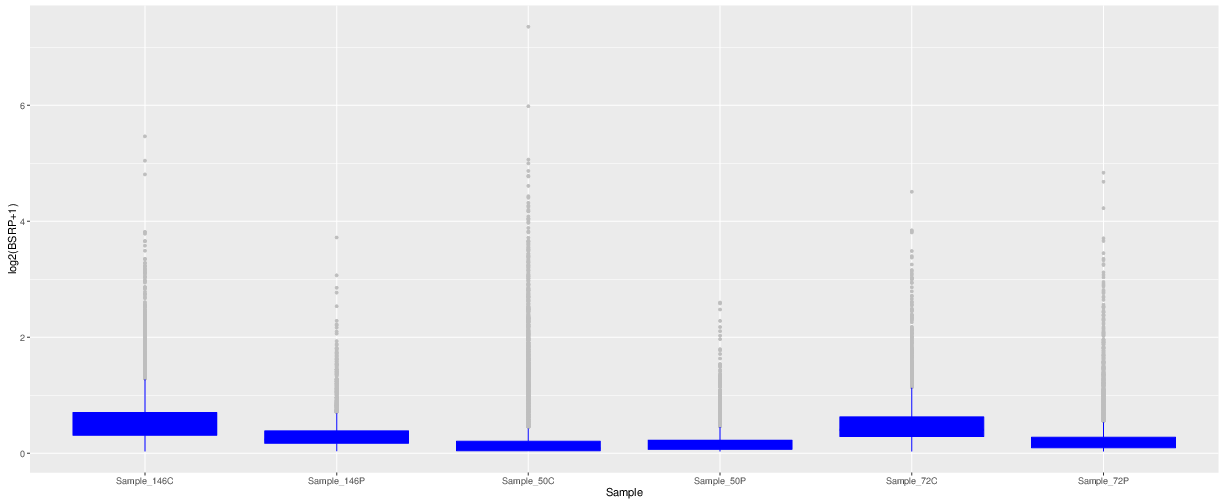

Supplement: Supplementary file 1 [file Data_Sheet_1.ZIP › Supplementary file/sample_cir_BSRP_boxplot.png]
